# Prognostic Significance of B7-H3, TROP2, and p16 Expression in Pleural Mesothelioma: Clinicopathological Correlations and Survival Outcomes

**DOI:** 10.3390/jcm15207998

**Published:** 2026-10-15

**Authors:** Neslihan Tas, Ramazan Oguz Yüceer, Tulay Koc, Yusuf Sönmez, Hasan Dogan

**Affiliations:** 1 Department of Pulmonology, Faculty of Medicine, Sivas Cumhuriyet University, 58140 Sivas, Turkey; dr.yusufsonmez@outlook.com; 2 Department of Pathology, Faculty of Medicine, Sivas Cumhuriyet University, 58140 Sivas, Turkey; r.yuceer66@hotmail.com (R.O.Y.); tkoc@cumhuriyet.edu.tr (T.K.); 3 Department of Thoracic Surgery, Faculty of Medicine, Sivas Cumhuriyet University, 58140 Sivas, Turkey; dr.18.hasandogan@gmail.com

**Keywords:** pleural mesothelioma, B7-H3, TROP2, p16, prognostic biomarkers, survival, antibody–drug conjugates

## Abstract

**Background:** Pleural mesothelioma (PM) is an aggressive malignancy with considerable biological heterogeneity and few established prognostic biomarkers. We evaluated the clinicopathological and prognostic relevance of B7-H3, TROP2, and p16 expression in PM. **Methods:** This retrospective study included 50 patients with histologically confirmed PM diagnosed between 2016 and 2025. Immunohistochemical expression of B7-H3, TROP2, and p16 was evaluated using an immunoreactivity scoring system. Associations with clinicopathological features were examined, and overall survival (OS) and progression-free survival (PFS) were analyzed using Kaplan–Meier and Cox proportional hazards methods. **Results:** High B7-H3, TROP2, and p16 expression was observed in 78%, 18%, and 20% of tumors, respectively. High B7-H3 expression was significantly associated with asbestos exposure, chemotherapy, and tumor stage. Patients with high B7-H3 expression had shorter median OS (11.0 vs. 42.0 months; *p* < 0.001) and PFS (11.0 vs. 40.0 months; *p* = 0.004) than those with negative/low expression. Neither TROP2 nor p16 expression was significantly associated with survival. In a parsimonious multivariable Cox model, high B7-H3 expression remained significantly associated with poorer OS after adjustment for disease stage (HR = 4.42, 95% CI: 1.29–15.21, *p* = 0.018). **Conclusions:** B7-H3 is frequently expressed in PM and was associated with poorer survival, including after adjustment for disease stage. These findings support further investigation of B7-H3 as a potential prognostic biomarker and therapeutic target in PM.

## 1. Introduction

Pleural mesothelioma (PM) is a rare, highly aggressive malignancy that arises from the mesothelial cells of the pleura. Although the incidence of asbestos exposure has declined in many countries, the long latency period between exposure and disease onset continues to impose a significant global health burden [1]. PM is characterized by diffuse pleural involvement, aggressive local invasion, and intrinsic resistance to conventional therapies, resulting in persistently poor clinical outcomes. Despite advances in multimodal treatment, including platinum-based chemotherapy, surgery in carefully selected patients, and more recently, immune checkpoint inhibitors, the median overall survival generally remains below two years [2].

In recent years, the treatment of PM has changed considerably, particularly following the introduction of immune checkpoint inhibitors. Nevertheless, only a subset of patients achieve durable responses, and disease progression remains common. Prognosis is generally estimated using established clinical and pathological factors, including histological subtype, disease stage, performance status, and treatment modality. However, these parameters do not fully account for the marked differences in clinical outcomes among patients with otherwise similar clinicopathological features [3]. This variability highlights the need for additional biomarkers that more closely reflect tumor biology, improve prognostic stratification, and potentially identify new therapeutic targets.

B7-H3 (CD276) has emerged as a biomarker of interest in thoracic malignancies. It belongs to the B7 family of immune regulatory molecules and is expressed at low levels in most normal tissues, while increased expression has been reported in several human cancers [4]. Although B7-H3 was initially studied mainly for its role in tumor immune escape, subsequent studies have shown that it may also contribute directly to tumor progression. B7-H3 has been linked to increased tumor cell proliferation, epithelial–mesenchymal transition, invasion, angiogenesis, metastasis, and resistance to apoptosis. In line with these biological effects, high B7-H3 expression has been associated with more aggressive tumor characteristics and poorer survival in a range of solid tumors [5,6,7].

The biological relevance of B7-H3 has also led to increasing interest in its potential as a therapeutic target. Several B7-H3-directed strategies, including monoclonal antibodies, antibody–drug conjugates (ADCs), bispecific antibodies, radionuclide-based therapies, and chimeric antigen receptor (CAR) T-cell therapies, have been clinically evaluated [8]. The phase I/II IDeate-PanTumor01 study evaluating the B7-H3-directed ADC ifinatamab deruxtecan (DS-7300a) demonstrated encouraging clinical activity and an acceptable safety profile in heavily pretreated patients with advanced solid tumors [9]. Similarly, other investigational agents, including enoblituzumab and vobramitamab duocarmazine (MGC018), are being evaluated in early phase clinical trials [10]. Although evidence specific to PM remains limited, patients with mesothelioma have been enrolled in several basket trials investigating B7-H3-targeted therapies, highlighting the increasing translational relevance of this immune checkpoint molecule in thoracic malignancies [11].

Another promising therapeutic target is trophoblast cell-surface antigen 2 (TROP2, encoded by TACSTD2), a transmembrane glycoprotein involved in regulating cell proliferation, migration, invasion, stemness, and metastatic potential through the activation of several oncogenic signaling pathways [12]. TROP2 overexpression has been reported in numerous epithelial cancers and is frequently associated with poor prognosis and resistance to treatment. More importantly, the successful clinical development of TROP2-directed ADCs has established this molecule as a leading therapeutic target in modern oncology [13]. Sacituzumab govitecan and datopotamab deruxtecan have demonstrated clinically meaningful efficacy in phase II and III trials involving breast cancer, non-small-cell lung cancer, and other epithelial malignancies [14]. These advances have stimulated growing interest in exploring TROP2-targeted therapies for less common thoracic tumors, including PM. Although dedicated clinical trials on mesothelioma are still lacking, ongoing basket studies, including patients with advanced PM, further emphasize the need to better define the biological and prognostic significance of TROP2 expression in this disease [15].

The tumor suppressor protein p16, encoded by the CDKN2A gene, is a key regulator of the G1/S cell cycle checkpoint through the inhibition of the CDK4/6–RB pathway. Homozygous deletion of CDKN2A is one of the hallmark genomic alterations in malignant pleural mesothelioma and has consistently been linked to more aggressive disease and unfavorable survival. Consequently, the assessment of CDKN2A deletion by fluorescence in situ hybridization (FISH) has become an important ancillary tool in the pathological diagnosis of mesothelioma [16]. In contrast, the prognostic value of p16 protein expression assessed by immunohistochemistry remains unclear. Previous studies have reported heterogeneous expression patterns and inconsistent associations with patient outcomes, leaving their clinical significance unresolved [17].

Emerging evidence suggests that the biological behavior of PM is determined by the interplay between immune escape mechanisms, oncogenic signaling pathways, and dysregulation of cell cycle control, rather than by alterations in a single molecular pathway. Accordingly, the simultaneous evaluation of biomarkers representing these complementary biological processes may provide a more comprehensive assessment of tumor biology than individual markers alone. Despite the increasing interest in B7-H3, TROP2, and p16, studies examining all three biomarkers within the same PM cohort remain scarce, and their combined prognostic significance has yet to be clearly established.

In this study, we evaluated the immunohistochemical expression of B7-H3, TROP2, and p16 in malignant pleural mesothelioma and investigated their relationships with clinicopathological features, overall survival, and progression-free survival. We also assessed the prognostic value of these markers, which are involved in different aspects of tumor biology, and their possible relevance to future biomarker-based treatment approaches.

## 2. Materials and Methods

This retrospective observational cohort study was conducted at the Department of Medical Pathology, Sivas Cumhuriyet University Faculty of Medicine, Sivas, Turkey. Consecutive patients diagnosed with malignant pleural mesothelioma (PM) between 1 January 2016, and 1 January 2025, were identified using the institutional pathology archive and hospital electronic medical record system. This study was designed and reported in accordance with the Strengthening the Reporting of Observational Studies in Epidemiology (STROBE) guidelines. Histopathological diagnoses were established according to the 2021 World Health Organization (WHO) Classification of Thoracic Tumors [18].

**Figure 1:**
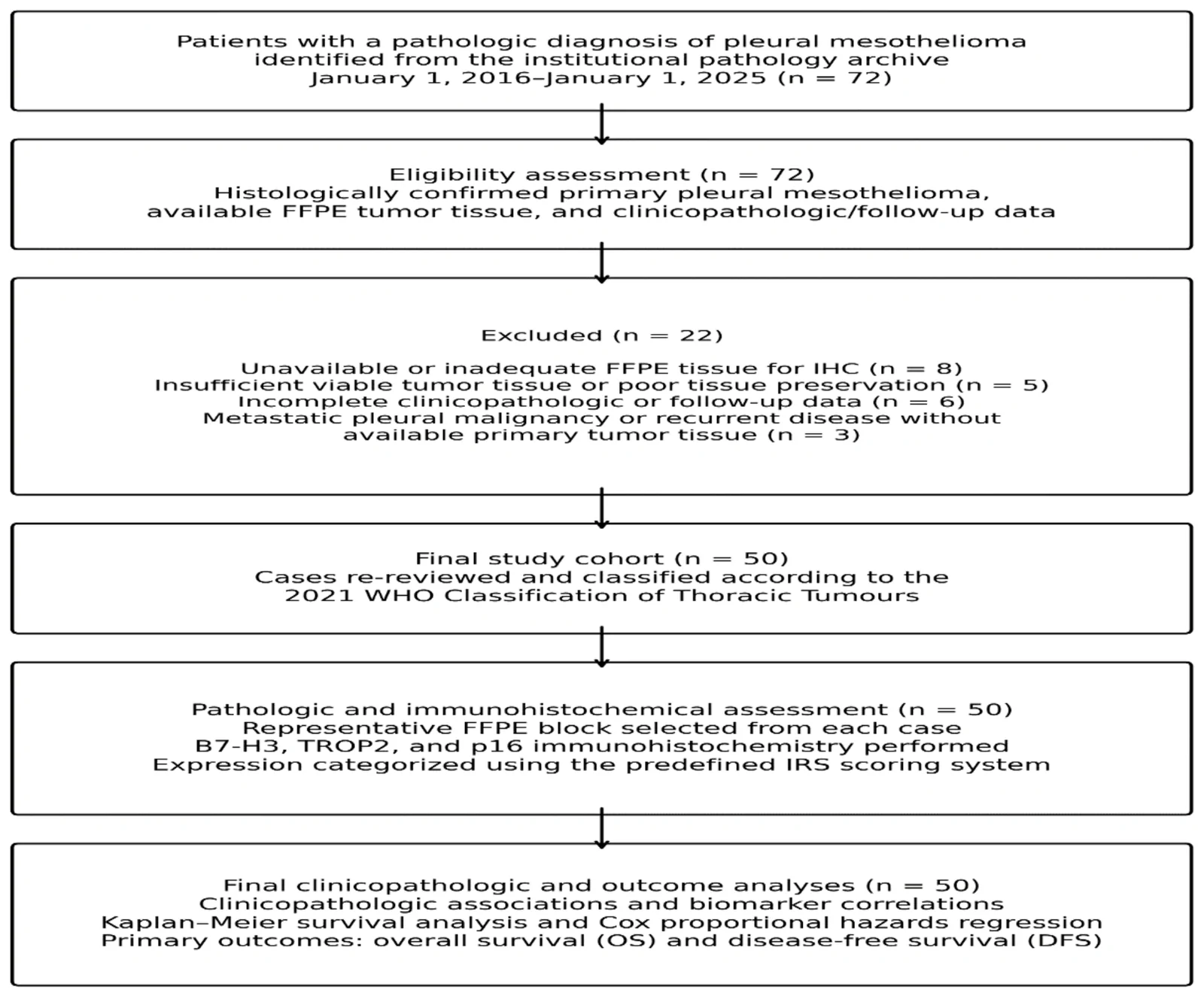
Patient selection and study flow.

Initially, 72 consecutive patients with a pathological diagnosis of PM were enrolled. Following application of the predefined eligibility criteria, 22 patients were excluded for one or more of the following reasons: unavailable or inadequate formalin-fixed paraffin-embedded (FFPE) tissue for immunohistochemical evaluation (n = 8), insufficient viable tumor tissue or poor tissue preservation (n = 5), incomplete clinicopathological or follow-up data (n = 6), and metastatic pleural malignancy or recurrent disease without available primary tumor tissue (n = 3). Consequently, 50 patients comprised the final study cohort (Figure 1).

### 2.1. Clinicopathological Data Collection

Demographic, clinical, pathological, treatment-related, and follow-up data were retrospectively retrieved from the hospital information system, pathology reports, oncology records, and the institutional cancer registry. The variables collected included age at diagnosis, sex, smoking history, history of asbestos exposure, comorbidities, Eastern Cooperative Oncology Group (ECOG) performance status when available, histological subtype, pathological stage according to the AJCC 8th edition TNM classification, treatment modality (surgery, chemotherapy, immunotherapy, and radiotherapy), recurrence status, date of recurrence, date of last follow-up, and survival status. Overall survival (OS) was defined as the interval between the date of pathological diagnosis and death from any cause or the last follow-up date. Progression-free survival (PFS) was calculated from the date of curative-intent surgery to the date of disease progression, defined as local recurrence or distant metastasis, or death from any cause. Patients without progression were censored at their last follow-up.

### 2.2. Histopathological Review

Archived H&E-stained slides and immunohistochemical stains were independently reviewed by two experienced pathologists (R.O.Y. and T.K.), both blinded to clinical characteristics, treatment data, and survival outcomes. Differences in immunohistochemical scoring were reviewed jointly and resolved by consensus. Histological subtypes were classified as epithelioid, biphasic, or sarcomatoid according to the current WHO diagnostic criteria. Additional pathological parameters, including nuclear atypia, mitotic activity, tumor necrosis, lymphovascular invasion, perineural invasion, and stromal characteristics, were reviewed. Cases with discordant interpretations were jointly re-examined using a multiheaded microscope until consensus was achieved.

### 2.3. Immunohistochemical Analysis

Representative FFPE tissue blocks containing the largest amount of viable tumor were selected for immunohistochemical analyses. Four-micrometer-thick sections were prepared, mounted on positively charged slides, deparaffinized with xylene, and rehydrated with graded ethanol. Heat-induced epitope retrieval was performed using citrate buffer (pH 6.0) or EDTA buffer (pH 9.0) according to the manufacturer’s recommendations for each antibody. Immunohistochemical staining was performed using a fully automated staining platform with a horseradish peroxidase-based polymer detection system and 3,3′-diaminobenzidine (DAB) as the chromogen. Appropriate positive and negative controls were included in each staining run to ensure the quality of the assay.

The sections were then stained with primary antibodies against Trop-2 (mouse monoclonal antibody, Clone B-9, 1:150 dilution, Santa Cruz Biotechnology, Santa Cruz, CA, USA), B7-H3 (rabbit monoclonal antibody, clone EPR20115, 1:4000 dilution, Abcam, Cambridge, UK), and p16INK4A (mouse monoclonal antibody, clone JC2, 1:100 dilution, Cell Marque, Rocklin, CA, USA). To ensure the accuracy of the staining procedure, positive controls for Trop-2 (breast epithelium), B7-H3 (prostate tissue), and p16 (cervical squamous cell carcinoma) were used, in accordance with the manufacturer’s instructions. All immunohistochemical staining procedures were performed using a fully automated immunohistochemistry device, the Roche Ventana Benchmark Ultra (Ventana Medical Systems, Inc., Roche Diagnostics, Tucson, AZ, USA).

Immunohistochemical evaluation was restricted to tumor cells, and stromal staining was excluded from scoring. For B7-H3 and TROP2, both membranous and cytoplasmic staining of tumor cells were considered. For p16, both nuclear and cytoplasmic staining of tumor cells were evaluated.

The immunoreactivity score (IRS) was calculated by combining the staining intensity and proportion of positive cells. Staining intensity was graded on a 4-point scale (0, no staining, 1 = weak; 2, moderate; 3, strong), and the extent of staining was scored according to the percentage of positive cells (0, 0%, 1 = <10%, 2 = 10–50%, 3 = >50%). The IRS was obtained by multiplying the intensity and extent scores, yielding a total score ranging from 0 to 9. For statistical analysis, expression levels were categorized as negative/low (IRS 0–4) or high (IRS > 4) [19,20].

### 2.4. Statistical Analysis

Statistical analyses were performed using IBM SPSS Statistics for Windows, version 27.0 (IBM Corp., Armonk, NY, USA), and R software version 4.5.2 (R Foundation for Statistical Computing, Vienna, Austria). Continuous variables were assessed for normality using the Shapiro–Wilk test and are presented as mean ± standard deviation (SD) or median (interquartile range [IQR]), as appropriate. Categorical variables are presented as frequencies and percentages.

Comparisons between categorical variables were performed using Pearson’s chi-square test or Fisher’s exact test, as appropriate. The correlations between biomarker expression levels were evaluated using Spearman’s rank correlation coefficient (ρ). Survival probabilities were estimated using the Kaplan–Meier method and compared using the log-rank test.

The prognostic significance of clinicopathological variables and biomarker expression was evaluated using Cox proportional hazard regression analysis. Variables were initially assessed using univariable Cox regression, and variables with a *p*-value < 0.10 were included in multivariable analyses. Three multivariable Cox regression models were constructed for both OS and PFS. Model 1 included baseline clinicopathological variables (age, sex, histological subtype, and tumor stage). Model 2 additionally incorporated treatment-related variables (surgery and chemotherapy). Model 3 further included the immunohistochemical biomarkers (B7-H3, TROP2, and p16) to determine their independent prognostic value after adjustment for clinicopathological and treatment factors. The proportional hazards assumption was assessed using Schoenfeld residuals. Given the limited sample size and potential risk of overfitting, an additional parsimonious multivariable Cox model including disease stage and B7-H3 expression was performed as a sensitivity analysis for OS. Hazard ratios (HRs) and 95% confidence intervals (CIs) were calculated for all the models. Statistical significance was set at *p* < 0.05.

## 3. Results

The 50 patients with pleural mesothelioma included in the study had a mean age of 66.7 ± 9.4 years (range, 44–85). Of these, 31 (62.0%) were men and 19 (38.0%) were women. Comorbidities were present in 27 patients (54.0%), 34 (68.0%) had a history of asbestos exposure, and 23 (46.0%) had a history of smoking.

Histopathological evaluation showed that the epithelioid subtype was predominant, accounting for 42 cases (84.0%), whereas the sarcomatoid and biphasic subtypes accounted for 6.0% and 10.0% of cases, respectively. Thirty-two patients (64.0%) received chemotherapy and six (12.0%) underwent surgical treatment. At diagnosis, 33 (66.0%) patients had stage I–II disease and 17 (34.0%) had stage III–IV disease.

High B7-H3 expression was observed in 39 patients (78.0%), whereas low or negative expression was detected in 11 patients (22.0%). B7-H3 expression was not significantly associated with age (*p* = 0.560), sex (*p* = 0.322), comorbidity (*p* = 0.380), histological subtype (*p* = 0.123), or surgical treatment (*p* = 0.604). However, significant associations were identified between asbestos exposure, smoking history, chemotherapy, and tumor stage. Patients with high B7-H3 expression more frequently had a history of asbestos exposure than those with low/negative expression (76.9% vs. 36.4%, *p* = 0.016). In contrast, smoking history was more common in the low/negative B7-H3 group (72.7% vs. 38.5%, *p* = 0.047), whereas non-smokers predominated among patients with high B7-H3 expression (61.5% vs. 27.3%). High B7-H3 expression was also associated with a higher rate of chemotherapy administration (71.8% vs. 36.4%, *p* = 0.037). Tumor stage demonstrated the strongest association with B7-H3 expression (*p* < 0.001), with stage I–II disease more frequently observed in the high-expression group (79.5%), whereas stage III–IV disease predominated in the low/negative expression group (81.8%).

High TROP2 expression was detected in nine patients (18.0%), whereas low/negative expression was observed in 41 patients (82.0%). TROP2 expression was not significantly associated with age (*p* = 0.642), sex (*p* = 0.068), comorbidity (*p* = 0.394), asbestos exposure (*p* = 0.605), histological subtype (*p* = 0.334), chemotherapy (*p* = 0.292), surgical treatment (*p* = 0.283), or tumor stage (*p* = 0.341). However, a significant association was identified between TROP2 expression and smoking history (*p* = 0.040). Smoking was more frequent among patients with high TROP2 expression than among those with low/negative expression (77.8% vs. 39.0%), whereas non-smokers were more common in the low/negative TROP2 expression group (61.0% vs. 22.2%). Although biphasic histology and chemotherapy administration were numerically more frequent in the high TROP2 group, these differences were not statistically significant.

**Figure 2:**
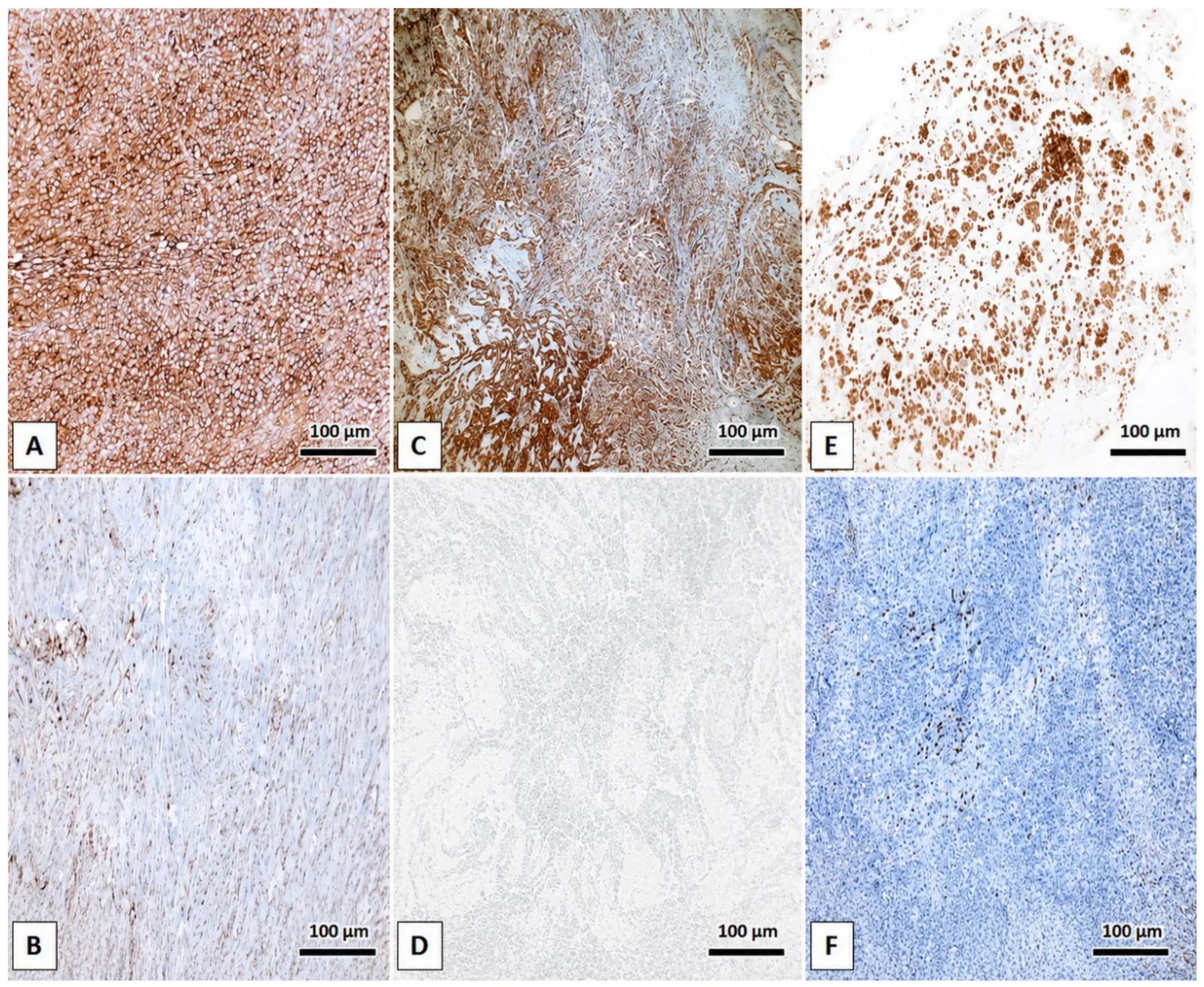
Representative immunohistochemical staining patterns of B7-H3, TROP2, and p16 in pleural mesothelioma. (**A**) High B7-H3 expression and (**B**) negative/low B7-H3 expression. (**C**) High TROP2 expression and (**D**) negative/low TROP2 expression. (**E**) High p16 expression and (**F**) negative/low p16 expression. Immunohistochemical evaluation was restricted to tumor cells, with stromal staining excluded from scoring. For B7-H3 and TROP2, membranous and cytoplasmic staining were evaluated, whereas both nuclear and cytoplasmic staining were considered for p16. DAB, 3,3′-diaminobenzidine. Original magnification, ×100; scale bars = 100 μm.

High p16 expression was identified in 10 patients (20.0%), whereas 40 patients (80.0%) were p16-negative/low. No significant associations were observed between p16 expression and age (*p* = 0.461), sex (*p* = 0.579), comorbidity (*p* = 0.526), asbestos exposure (*p* = 0.400), smoking history (*p* = 0.261), histological subtype (*p* = 0.180), chemotherapy (*p* = 0.479), surgical treatment (*p* = 0.656), or tumor stage (*p* = 0.204). Although p16-positive tumors showed numerically higher proportions of smoking history (60.0% vs. 42.5%), biphasic histology (20.0% vs. 7.5%), chemotherapy administration (70.0% vs. 62.5%), and advanced-stage disease (50.0% vs. 30.0%) than p16-negative tumors, none of these differences reached statistical significance (Table 1, Figure 2).

Spearman’s correlation analysis revealed no significant correlations among the B7-H3, TROP2, and p16 expression levels. B7-H3 expression was not correlated with TROP2 (ρ = −0.003, *p* = 0.986) or p16 (ρ = −0.217, *p* = 0.130) expression. Similarly, no significant correlation was observed between TROP2 and p16 expression levels (ρ = 0.026, *p* = 0.858). Overall, the three biomarkers exhibited independent expression patterns in this study cohort.

Among the 50 patients, 41 deaths (82.0%) occurred during follow-up, while 9 patients (18.0%) were censored for overall survival (OS). For progression-free survival (PFS), 43 events (86.0%) were observed, while 7 patients (14.0%) were censored. The median follow-up duration, estimated using the reverse Kaplan–Meier method, was 51 months.

**Figure 3:**
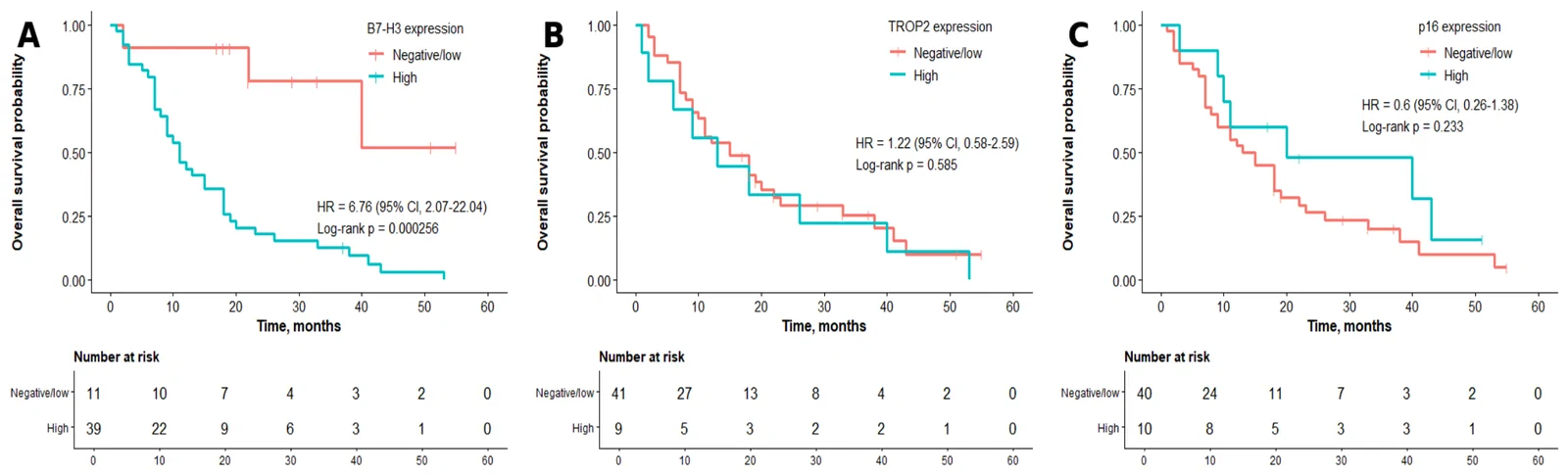
Kaplan–Meier curves for overall survival (OS) according to B7-H3, TROP2, and p16 expression in pleural mesothelioma. (**A**) OS according to B7-H3 expression (negative/low vs. high); (**B**) OS according to TROP2 expression (negative/low vs. high); and (**C**) OS according to p16 expression (negative/low vs. high). The numbers at risk are shown below each Kaplan–Meier curve. *p*-values were calculated using the log-rank test. OS, overall survival.

Patients with low/negative B7-H3 expression had a significantly longer median overall survival than those with high B7-H3 expression (42.0 months [95% confidence interval (CI): 30.2–53.8] vs. 11.0 months [95% CI: 7.3–14.7]; *p* < 0.001). In contrast, TROP2 expression was not associated with overall survival, with median survival times of 15.0 months (95% CI: 7.9–22.1) in the low/negative group and 13.0 months (95% CI: 1.3–24.7) in the high-expression group (*p* = 0.585). Similarly, p16 expression was not significantly associated with overall survival. The median overall survival was 13.0 months (95% CI: 6.8–19.2) in p16-negative/low patients and 20.0 months (95% CI: 0.0–53.3) in p16-high patients (*p* = 0.233) (Figure 3).

**Figure 4:**
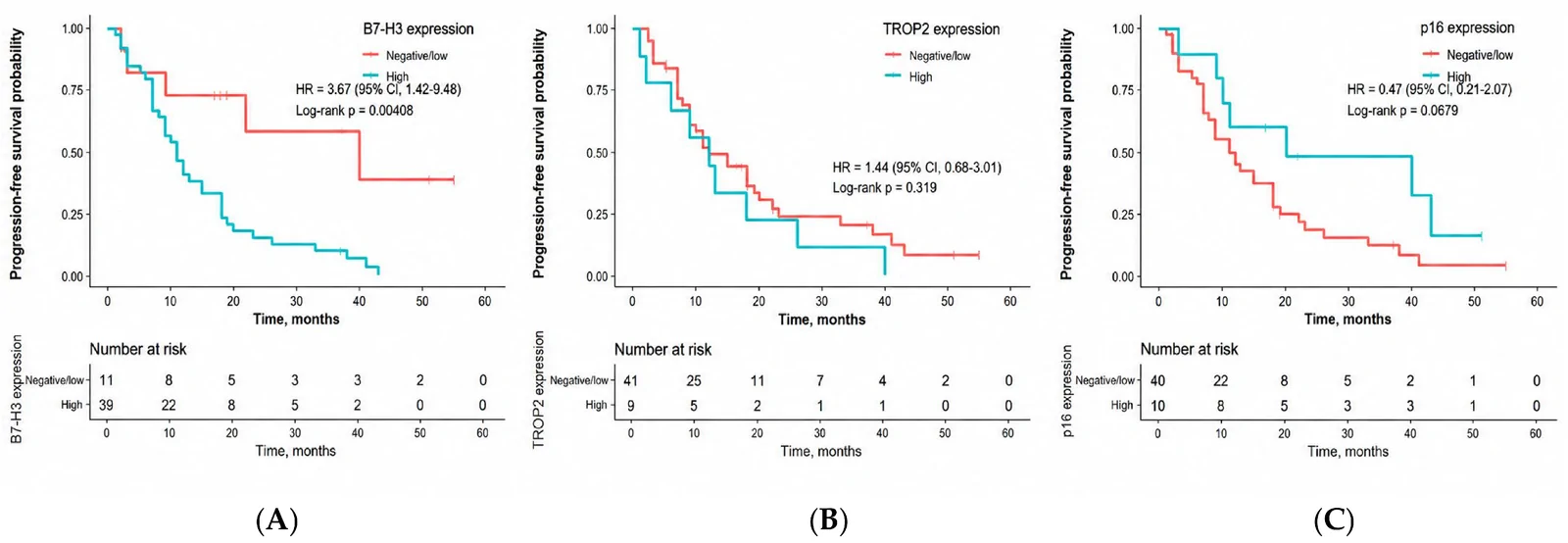
Kaplan–Meier curves for progression-free survival (PFS) according to B7-H3, TROP2, and p16 expression in pleural mesothelioma. (**A**) PFS according to B7-H3 expression (negative/low vs. high); (**B**) PFS according to TROP2 expression (negative/low vs. high); and (**C**) PFS according to p16 expression (negative/low vs. high). The numbers at risk are shown below each Kaplan–Meier curve. *p*-values were calculated using the log-rank test. PFS, progression-free survival.

Patients with low/negative B7-H3 expression had a significantly longer median progression-free survival than those with high B7-H3 expression (40.0 months, 95% CI: 4.7–75.3 vs. 11.0 months, 95% CI: 7.9–14.1; *p* = 0.004). In contrast, TROP2 expression was not associated with progression-free survival, with identical median survival times of 12.0 months in both the low/negative and high-expression groups (95% CI: 6.8–17.2 vs. 3.2–20.8 months, *p* = 0.319). Similarly, p16 expression was not significantly associated with progression -free survival. The median survival was 11.0 months (95% CI: 6.4–15.6) in p16-negative/low patients and 20.0 months (95% CI: 0.0–53.3) in p16-high patients (*p* = 0.680) (Figure 4).

The results of the univariate Cox regression analyses are summarized in Table 2 For overall survival (OS), histological subtype (HR = 1.622, 95% CI: 1.033–2.548, *p* = 0.036), tumor stage (HR = 0.099, 95% CI: 0.037–0.265, *p* < 0.001), and B7-H3 expression (HR = 7.014, 95% CI: 2.138–23.011, *p* = 0.001) were significantly associated with survival, whereas age, sex, comorbidity, asbestos exposure, smoking history, surgery, TROP2, and p16 were not. For PFS, only tumor stage (HR = 0.040, 95% CI: 0.009–0.177, *p* < 0.001) and B7-H3 expression (HR = 3.580, 95% CI: 1.386–9.245, *p* = 0.008) were significant predictors, while histological subtype showed a non-significant trend (HR = 1.476, 95% CI: 0.945–2.305, *p* = 0.087). No significant associations were observed between the remaining clinicopathological variables or biomarkers (Table 2).

### Multivariable Cox Regression Analysis for Overall Survival

Three multivariable Cox proportional hazards models were constructed to evaluate independent prognostic factors for overall survival (Table 3). Model 1 included age, sex, histological subtype, and disease stage. Advanced-stage disease (stage III–IV) was independently associated with an increased risk of death compared with early-stage disease (stage I–II) (HR = 10.75, 95% CI: 3.92–29.41, *p* < 0.001). Age, sex, and histological subtype were not independently associated with overall survival.

Model 2 additionally incorporated treatment variables (surgery and chemotherapy). Advanced-stage disease remained independently associated with an increased risk of death (HR = 10.87, 95% CI: 3.85–31.25, *p* < 0.001), whereas neither surgery nor chemotherapy showed a significant association with overall survival.

In the fully adjusted model (Model 3), which further included TROP2, p16, and B7-H3 expression, advanced-stage disease remained independently associated with poorer overall survival (HR = 7.75, 95% CI: 2.48–23.81, *p* < 0.001). High B7-H3 expression was also independently associated with an increased risk of death (HR = 7.81, 95% CI: 1.68–36.36, *p* = 0.009). No significant associations with overall survival were observed for TROP2 or p16 expression (Table 3).

Given the limited sample size, a parsimonious multivariable Cox regression model including disease stage and B7-H3 expression was additionally performed. In this model, advanced-stage disease (stage III–IV) was independently associated with an increased risk of death (HR = 8.42, 95% CI: 3.03–23.38, *p* < 0.001). High B7-H3 expression also remained independently associated with poorer overall survival after adjustment for disease stage (HR = 4.42, 95% CI: 1.29–15.21, *p* = 0.018) (Table 4).

Three multivariable Cox proportional hazards models were constructed to evaluate independent prognostic factors for progression-free survival (PFS) (Table 5). In Model 1, which included age, sex, histological subtype, and disease stage, advanced-stage disease (stage III–IV) was independently associated with a significantly increased risk of progression or death compared with stage I–II disease (HR = 29.39, 95% CI: 6.26–137.97, *p* < 0.001). Age, sex, and histological subtype were not significantly associated with PFS.

After the addition of surgery and chemotherapy in Model 2, advanced stage remained independently associated with poorer PFS (HR = 31.21, 95% CI: 6.53–149.12, *p* < 0.001), whereas neither treatment variable showed a significant association with PFS.

In the fully adjusted Model 3, which additionally included TROP2, p16, and B7-H3 expression, advanced stage remained the only independent prognostic factor for PFS (HR = 25.18, 95% CI: 4.58–138.35, *p* < 0.001). TROP2, p16, and B7-H3 expression were not independently associated with PFS.

## 4. Discussion

The present study demonstrates that B7-H3 is the most clinically relevant biomarker among the three markers evaluated for pleural mesothelioma. High B7-H3 expression was observed in 78% of tumors and was independently associated with significantly shorter overall survival after adjustment for established clinicopathological variables. In contrast, neither TROP2 nor p16 expression was independently associated with survival, and no significant correlations were identified among the three biomarkers, suggesting that they reflect distinct biological processes rather than a shared molecular pathway.

The high frequency of B7-H3 expression observed in our cohort is consistent with the recent Mesoscape study, in which membranous B7-H3 (CD276) expression was reported in approximately 86% of 353 pleural mesotheliomas [11]. In contrast to that study, however, high B7-H3 expression in our cohort was independently associated with poorer overall survival. Several methodological and cohort-related factors may account for this difference. The use of whole tissue sections rather than tissue microarrays, differences in scoring systems and cutoff values, and variation in patient and tumor characteristics may affect the assessment of B7-H3 and its association with outcome. In addition, intratumoral heterogeneity of B7-H3 expression has been reported in pleural mesothelioma and may partly explain the differences in prognostic findings between studies [11].

The association between B7-H3 expression and poor survival is consistent with its known biological functions. In addition to its role in immune regulation, B7-H3 has been implicated in epithelial–mesenchymal transition, angiogenesis, tumor invasion, and resistance to apoptosis [4,21]. These effects may be relevant in pleural mesothelioma, where an immunosuppressive tumor microenvironment contributes to disease progression and treatment resistance. In our cohort, the association between B7-H3 expression and overall survival persisted after adjustment for clinicopathological factors, suggesting that B7-H3 may have potential prognostic value. However, the relatively small sample size and uncertainty surrounding the effect estimate warrant cautious interpretation and validation in larger independent cohorts.

Interestingly, high B7-H3 expression was more common in early-stage tumors despite its association with poorer survival. One possible explanation is that B7-H3 expression reflects aggressive tumor biology that is not captured by anatomic stage alone. However, this finding should be interpreted cautiously, particularly given the relatively small number of tumors with low B7-H3 expression. Confirmation in larger, independent cohorts is needed to determine whether this association is reproducible.

The frequent expression of B7-H3 in pleural mesothelioma may also have therapeutic relevance. Several B7-H3–directed approaches, including antibody-drug conjugates, monoclonal and bispecific antibodies, and CAR T-cell therapies, are currently under clinical investigation [9,22]. However, the prognostic association observed in the present study should be distinguished from the potential predictive value of B7-H3 for response to B7-H3-directed therapies. Treatment response to B7-H3-targeted agents was not evaluated in our cohort, and our findings therefore do not establish B7-H3 as a predictive biomarker. Prospective studies will be needed to determine whether B7-H3 expression can identify patients who are more likely to benefit from B7-H3–directed therapies.

High TROP2 expression was detected in only 18% of tumors, closely resembling the approximately 20% prevalence reported by Avilés-Salas et al. [23]. However, unlike in that study, we found no association between TROP2 expression and survival. A previous study demonstrated that TROP2 positivity independently predicted shorter progression-free and overall survival. Several methodological differences, including sample size, antibody selection, scoring criteria, and patient characteristics, may explain these discrepant findings. Only nine patients in our cohort had high TROP2 expression, limiting our ability to assess its association with survival. Nevertheless, the presence of TROP2-positive tumors across independent mesothelioma cohorts supports continued investigation of TROP2 as a potential therapeutic target, particularly in the context of TROP2-directed antibody-drug conjugates.

We also observed an association between TROP2 expression and smoking history. This finding should be interpreted cautiously, as there is currently no direct evidence linking smoking exposure to increased TROP2 expression in mesothelioma. Given the small number of TROP2-high tumors, this association may reflect limited sample size or unmeasured confounding and requires confirmation in independent cohorts.

Retained p16 expression was observed in one-fifth of the tumors but was not associated with clinicopathological characteristics or survival. This finding does not necessarily conflict with previous studies that demonstrated poorer outcomes in tumors with CDKN2A deletion. Immunohistochemical p16 expression is an imperfect surrogate for CDKN2A genomic status, and its prognostic performance is inferior to that of the combined assessment with MTAP immunohistochemistry or CDKN2A fluorescence in situ hybridization [24,25]. Therefore, the absence of prognostic significance in our cohort likely reflects the limited ability of p16 immunohistochemistry alone to fully characterize the alterations in the CDKN2A pathway.

We found no significant correlations among B7-H3, TROP2, and p16 expression. Given the limited sample size, the absence of statistically significant correlations should not be interpreted as evidence that these biomarkers are biologically independent or necessarily represent distinct molecular pathways. Further studies incorporating molecular and functional data are needed to determine whether these pathways define distinct tumor subgroups and whether they have implications for biomarker-based treatment selection.

Several limitations should be considered when interpreting our findings. The retrospective, single-center design and relatively small cohort may limit the generalizability of the results. In addition, although the biomarker cutoffs were selected based on previous literature, their applicability should be further validated in independent cohorts. Molecular data on CDKN2A status were not available, and the relationship between biomarker expression and treatment response could not be fully assessed. Nevertheless, all three biomarkers were evaluated using a consistent methodology in a well-characterized cohort with long-term follow-up, allowing their clinicopathological and prognostic associations to be assessed within the same patient population.

## 5. Conclusions

In conclusion, B7-H3 showed the strongest prognostic association among the three biomarkers evaluated in this cohort and was associated with poorer overall survival, whereas TROP2 and p16 were not independently associated with survival. The frequent expression of B7-H3 and its association with outcome support further investigation of its potential prognostic value in pleural mesothelioma. Larger, independent studies are needed to validate these findings. Moreover, prospective studies evaluating B7-H3-directed therapies are required to determine whether B7-H3 expression has predictive value for treatment response.

## Figures and Tables

**Table 1:** Association of TROP2, p16 and B7-H3 expression with clinicopathological characteristics.

Variable	Category	B7-H3 Negative/Low n (%)	B7-H3 High n (%)	*p*	TROP2 Negative/Low n (%)	TROP2 High n (%)	*p*	p16-Negative/Low n (%)	p16-High n (%)	*p*
Age	<65 years	4 (36.4)	13 (33.3)	0.560	14 (34.1)	3 (33.3)	0.642	13 (32.5)	4 (40.0)	0.461
	≥65 years	7 (63.6)	26 (66.7)		27 (65.9)	6 (66.7)		27 (67.5)	6 (60.0)	
Sex	Female	3 (27.3)	16 (41.0)	0.322	18 (43.9)	1 (11.1)	0.068	15 (37.5)	4 (40.0)	0.579
	Male	8 (72.7)	23 (59.0)		23 (56.1)	8 (88.9)		25 (62.5)	6 (60.0)	
Comorbidity	No	6 (54.5)	17 (43.6)	0.380	18 (43.9)	5 (55.6)	0.394	18 (45.0)	5 (50.0)	0.526
	Yes	5 (45.5)	22 (56.4)		23 (56.1)	4 (44.4)		22 (55.0)	5 (50.0)	
Asbestos exposure	No	7 (63.6)	9 (23.1)	0.016	13 (31.7)	3 (33.3)	0.605	12 (30.0)	4 (40.0)	0.400
	Yes	4 (36.4)	30 (76.9)		28 (68.3)	6 (66.7)		28 (70.0)	6 (60.0)	
Smoking history	No	3 (27.3)	24 (61.5)	0.047	25 (61.0)	2 (22.2)	0.040	23 (57.5)	4 (40.0)	0.261
	Yes	8 (72.7)	15 (38.5)		16 (39.0)	7 (77.8)		17 (42.5)	6 (60.0)	
Histological subtype	Epithelioid	11 (100.0)	31 (79.5)	0.123	35 (85.4)	7 (77.8)	0.334	35 (87.5)	7 (70.0)	0.180
	Sarcomatoid	0 (0.0)	3 (7.7)		3 (7.3)	0 (0.0)		2 (5.0)	1 (10.0)	
	Biphasic	0 (0.0)	5 (12.8)		3 (7.3)	2 (22.2)		3 (7.5)	2 (20.0)	
Chemotherapy	No	7 (63.6)	11 (28.2)	0.037	16 (39.0)	2 (22.2)	0.292	15 (37.5)	3 (30.0)	0.479
	Yes	4 (36.4)	28 (71.8)		25 (61.0)	7 (77.8)		25 (62.5)	7 (70.0)	
Surgery	No	10 (90.9)	34 (87.2)	0.604	35 (85.4)	9 (100.0)	0.283	35 (87.5)	9 (90.0)	0.656
	Yes	1 (9.1)	5 (12.8)		6 (14.6)	0 (0.0)		5 (12.5)	1 (10.0)	
Stage	I–II	2 (18.2)	31 (79.5)	<0.001	26 (63.4)	7 (77.8)	0.341	28 (70.0)	5 (50.0)	0.204
	III–IV	9 (81.8)	8 (20.5)		15 (36.6)	2 (22.2)		12 (30.0)	5 (50.0)	

**Table 2:** Univariable Cox regression analyses for overall survival and progression-free survival.

Variable	Overall Survival HR (95% CI)	*p*	Progression-Free Survival HR (95% CI)	*p*
Age group	0.865 (0.456–1.644)	0.659	1.057 (0.561–1.989)	0.864
Sex	0.965 (0.513–1.815)	0.911	1.067 (0.573–1.988)	0.839
Comorbidity	1.135 (0.611–2.110)	0.688	0.933 (0.510–1.705)	0.821
Asbestos exposure	1.483 (0.741–2.970)	0.266	1.110 (0.577–2.135)	0.755
Smoking history	0.600 (0.315–1.142)	0.120	0.628 (0.336–1.175)	0.146
Histological subtype	1.622 (1.033–2.548)	0.036	1.476 (0.945–2.305)	0.087
Tumor stage	0.099 (0.037–0.265)	<0.001	0.040 (0.009–0.177)	<0.001
Chemotherapy	0.523 (0.108–1.280)	0.808	0.808 (0.422–1.549)	0.521
Surgery	0.594 (0.211–1.675)	0.325	0.513 (0.182–1.445)	0.206
B7-H3 expression	7.014 (2.138–23.011)	0.001	3.580 (1.386–9.245)	0.008
TROP2 expression	1.454 (0.689–3.068)	0.325	1.440 (0.686–3.020)	0.335
p16 expression	0.549 (0.238–1.268)	0.160	0.478 (0.208–1.095)	0.081

**Table 3:** Multivariable Cox regression analysis for overall survival.

Variable	Model 1 HR (95% CI)	*p*	Model 2 HR (95% CI)	*p*	Model 3 HR (95% CI)	*p*
Age	0.98 (0.95–1.02)	0.334	0.98 (0.95–1.02)	0.389	0.99 (0.95–1.03)	0.633
Male sex	1.14 (0.59–2.20)	0.702	1.09 (0.56–2.15)	0.794	1.64 (0.74–3.63)	0.219
Sarcomatoid histology	2.13 (0.61–7.46)	0.238	2.72 (0.67–11.08)	0.162	1.05 (0.22–4.98)	0.948
Biphasic histology	1.20 (0.45–3.21)	0.719	1.12 (0.42–3.04)	0.818	1.25 (0.42–3.69)	0.691
Stage III–IV	10.75 (3.92–29.41)	<0.001	10.87 (3.85–31.25)	<0.001	7.75 (2.48–23.81)	<0.001
Surgery	–	–	0.92 (0.29–2.97)	0.895	0.61 (0.16–2.23)	0.452
Chemotherapy	–	–	1.38 (0.62–3.08)	0.425	0.56 (0.19–1.68)	0.301
TROP2 high	–	–	–	–	0.90 (0.36–2.25)	0.818
p16 positive	–	–	–	–	0.75 (0.26–2.18)	0.603
B7-H3 high	–	–	–	–	7.81 (1.68–36.36)	0.009

Reference categories: female sex; epithelioid histology; stage I–II; no surgery; no chemotherapy; low/negative TROP2; negative p16; and low/negative B7-H3 expression.

**Table 4:** Parsimonious multivariable Cox regression analysis for overall survival.

Variable	HR (95% CI)	*p*
Advanced stage (III–IV)	8.42 (3.03–23.38)	<0.001
B7-H3 high expression	4.42 (1.29–15.21)	0.018

Reference categories: early-stage disease (stage I–II) for disease stage and low/negative expression for B7-H3.

**Table 5:** Multivariable Cox regression analysis for progression-free survival.

Variable	Model 1 HR (95% CI)	*p*	Model 2 HR (95% CI)	*p*	Model 3 HR (95% CI)	*p*
Age	0.99 (0.95–1.02)	0.455	0.99 (0.95–1.02)	0.390	0.99 (0.96–1.03)	0.650
Male sex	1.30 (0.68–2.50)	0.428	1.86 (0.80–4.34)	0.149	1.82 (0.76–4.35)	0.180
Sarcomatoid histology	1.46 (0.42–5.01)	0.549	0.81 (0.18–3.55)	0.779	0.78 (0.16–3.73)	0.759
Biphasic histology	0.79 (0.29–2.16)	0.644	0.86 (0.31–2.38)	0.775	0.95 (0.32–2.81)	0.920
Stage III–IV	29.39 (6.26–137.97)	<0.001	31.21 (6.53–149.12)	<0.001	25.18 (4.58–138.35)	<0.001
Surgery	–	–	0.74 (0.24–2.28)	0.601	0.67 (0.20–2.31)	0.531
Chemotherapy	–	–	0.53 (0.21–1.35)	0.183	0.50 (0.17–1.44)	0.200
TROP2 high	–	–	–	–	1.18 (0.48–2.92)	0.717
p16 positive	–	–	–	–	0.62 (0.24–1.65)	0.340
B7-H3 high	–	–	–	–	1.35 (0.35–5.26)	0.666

Reference categories: female sex; epithelioid histology; stage I–II; no surgery; no chemotherapy; low/negative TROP2; negative p16; and low/negative B7-H3 expression.

## Data Availability

The data presented in this study are available upon request from the corresponding author due to privacy restrictions.

## Abbreviations

ADC	Antibody–drug conjugate
AJCC	American Joint Committee on Cancer
CAR	Chimeric antigen receptor
CDKN2A	Cyclin-dependent kinase inhibitor 2A
CI	Confidence interval
DAB	3,3′-Diaminobenzidine
PFS	Progression-free survival
ECOG	Eastern Cooperative Oncology Group
FFPE	Formalin-fixed paraffin-embedded
FISH	Fluorescence in situ hybridization
H&E	Hematoxylin and eosin
HR	Hazard ratio
IHC	Immunohistochemistry
IQR	Interquartile range
IRS	Immunoreactivity score
OS	Overall survival
PM	Pleural mesothelioma
RB	Retinoblastoma protein
SD	Standard deviation
STROBE	Strengthening the Reporting of Observational Studies in Epidemiology
TNM	Tumor–node–metastasis
TROP2	Trophoblast cell-surface antigen 2
WHO	World Health Organization
